# Unmet needs for contraception: A comparative study among Somali immigrant women in Oslo and their original population in Mogadishu, Somalia

**DOI:** 10.1371/journal.pone.0220783

**Published:** 2019-08-15

**Authors:** Abdi A. Gele, Fathia K. Musse, Samera Qureshi

**Affiliations:** 1 Department of Health Service Research, Norwegian Institute of Public Health, Oslo, Norway; 2 Department of Family Counselling, Oslo Municipality, Oslo, Norway; 3 Department of Health Service Research, Norwegian Institute of Public Health, Oslo, Norway; Queen's University at Kingston, CANADA

## Abstract

**Introduction:**

Unmet need for contraception is defined as the proportion of fertile individuals who do not use contraceptives despite wanting to space or limit their childbearing. Studies show that immigrant women in Europe, have higher rates of unintended pregnancies and abortion than native born women. Somali women, have the highest fertility rate in Norway which is much higher than the total fertility rate in Norway (4.0 vs. 1.7). This study investigates the unmet need for contraception among Somali immigrant women in Oslo, Norway, compared to their original population in Mogadishu, Somalia.

**Methods:**

A community based, cross sectional study was carried out among Somali women in Oslo (N = 228) and Mogadishu (N = 229) from May to December 2018. Pre-structured questionnaires were given to women who were recruited through snow-ball sampling. Data was analyzed using SPSS version 25. We performed a chi-square test for the analyses of categorical variables, a *t*-test for continuous variables and multivariate logistic analysis to determine the association between exposure and outcome variable.

**Results:**

The unmet needs for contraception among Somali women in Oslo was 20.2%, which is similar to unmet needs for contraception of women in many sub-Saharan African countries. The unmet needs for Somali immigrant women in Oslo (20.2) is two times lower than that of their original population in Somalia (48.5). The odds of having unmet needs for contraception was nearly, three times higher among Somali women in Mogadishu compared to those in Oslo (OR: 2.6, CI: 2.56–7.68). The mean intended fertility was 4 among the women in Oslo and 10.8 in Mogadishu. About 13.4% of study participants in Oslo and 86.6% of those in Mogadishu consider modern contraception irrelevant for women’s health. Nearly 50% of women in both places had unintended childbirth on one or more occasion.

**Conclusion:**

The study results show the prevalence of unmet needs for contraception among Somali immigrant women Oslo, is 4 fold higher than that of Norway (20.2 vs 5.5). Information Education Communication to both men and women, may reduce the high unmet need for contraception and also improve partner communication on family planning among Somali immigrants in Oslo. Training primary health providers for provision of tailored information about the modern contraception to immigrant women, which includes an individualized counselling may improve partners’ knowledge, demand and uptake of modern contraception.

## Background

Currently, about 214 million women across the world have an unmet need for modern contraceptives [1]. As a result, over 85 million mistimed or unwanted pregnancies occur annually in the world, contributing to high rates of induced abortions, maternal morbidity and mortality [2]. Unmet need is defined as the proportion of fertile individuals who do not use contraceptive methods despite they want to space or limit their childbearing. The European Action Plan for Sexual and Reproductive Health emphasizes the importance of improving access to contraceptive services for immigrants [3]. Nonetheless, studies show that immigrant women in Europe, have higher rates of unintended pregnancies and abortion than native born women [4–8], raising concerns about their access to quality contraceptive education and healthcare [9, 10].

Research conducted in Scandinavia specifically has also highlighted that immigrant women seek induced abortion in higher numbers than would be expected given their proportion of the population [7, 10, 11]. In Finland, over 73% of married women with Somali descent were reported as not using contraception [12]. In Sweden, a study found that 57% of immigrant women seeking abortion did not use any contraceptive at the time of conception [7]. In Norway, a study found that 25% of women seeking abortion were immigrants from non-Western countries [10]. Further, a register data analysis in Norway found that immigrant groups are 47–71% less likely to use hormonal contraception than their native counterparts [13], which may result in high rates of unintended pregnancies and abortion among immigrants. Preventing unintended pregnancies, which include pregnancies occurring sooner than desired and those that were not wanted at all, is fundamental in achieving the health related, UN sustainable Development goals [14, 15].

Approximately 43,000 Somali immigrants live in Norway, thereby constituting the largest non-western immigrant group in the country, and the largest African community in Norway, Finland, Sweden and Denmark. Somalia has the second highest fertility rate in the world (6.2 births per woman), which exceeds fertility rate in sub-Saharan Africa (4.8 births per woman) and the world (2.4 births per woman) [16]. In Somali culture, a woman’s status is enhanced by the number of children she produces. Therefore, Somali men and women equate using modern contraception to voluntary infertility, which sharply contrasts with the Somali culture of reproduction. Consequently, not only do a striking majority of Somali women prefer having many children, but knowledge of modern contraception is low for couples, including even those who are most motivated to avoid pregnancy [17].

Cultural and religious norms were found to have an important influence on Somali immigrants’ way of life in the USA, and these norms largely impede the uptake of reproductive health knowledge due to stigma and fear of judgment [18]. Somali immigrants bring their believes and attitudes toward contraception with them during migration. For instance, the main reason for not using contraception among Somali immigrant women in Finland was reported to be a religious reason [12], which is exactly similar with the reasons presented by women in Somalia, where using contraceptives with the intention to limit the number of children is considered to be against Islamic values and practices [19]. This highlights that the culture of immigrant’s country of origin may shape their contraceptive use regardless of availability of the methods. Therefore, a comparative study between immigrants and their original population may provide vital information about how much immigrants have sustained or changed their attitudes and believes toward contraception after migration.

In Somalia, the most available contraception methods are injection and pills, and it is mainly found in private clinics with variable costs. There are few areas where NGOs provide long acting reversible contraception (LARC) to women for free. The utilization of contraceptives depends on the communication between the health provider and the user. For example, when the term ‘birth spacing’ is used by the provider, contraception is accepted by Somali men and women. However, when the term ‘family planning’ is used, contraception is rejected as it is perceived to contradict the moral standards of childbearing accepted by Islamic religion [19]. The most popular child spacing methods in Somalia are traditional methods such as breastfeeding and coitus interruptus. The need for modern contraception must be justified, and the prescription of injectable contraceptives and LARC require the approval of the husband. Methods such as condoms are perceived to promote extra-marital sex and is therefore prohibited [19]

Contrastingly, the Norwegian healthcare system is predominantly public and everyone is entitled to have a general practitioner (GP) who can prescribe contraceptives and insert implants and intrauterine devices (IUDs). There is co-payment for the GP visit, while midwives and nurses can also prescribe contraceptives and they are free of charge. Contraceptive methods are subsidized partly or wholly for women under the age of 22 while older women must pay the full cost of the method. The utilization of reproductive services depends on language proficiency, and comprehension of information received about the health system. Doctor-patient interaction patterns, and language and cultural differences between immigrants and health providers are critical in immigrant’s access to services [20].

While Somali women in Norway experience varying degrees of success in integration to the Norwegian culture, and a change in attitudes related to harmful traditional practices such as FGM have been observed [21], they sustain many other traditions [4]. According to the Statistics Norway, women from Somalia, have the highest fertility rate in Norway which is much higher than the total fertility rate in Norway (4 vs 1.7) [22]. However, it is unclear whether the high fertility rate among Somali women in Norway is contributed by high rates of unintended pregnancies. Improving reproductive health of immigrant women is receiving growing attention in Norway, but the access and utilization to modern contraception and interventions to prevent unintended pregnancies remain a hugely under-researched area.

## Materials and methods

A community based, cross sectional study was carried out to investigate the unmet need for contraception among Somali immigrant women in Oslo, Norway, compared to their original population in Mogadishu, Somalia. Pre-tested structured questionnaires were consecutively administered to 228 Somali immigrant women in Oslo and 229 Somali women in Mogadishu from May to December 2018. Nearly 50% of Somali immigrants in Norway are estimated to live in Oslo area. Mogadishu is the capital of Somalia with a population of two million and the former home of many Somali immigrants who live in Norway. Norwegian Regional Committee for medical and health research ethics (REK) approved the study with approval number: 2017/2386. Oral consent were obtained from all participants in the study. The reason was the fact that most participants in Somalia, and many in Norway were illiterate

Only married women were included in the study in both settings. The questionnaires were developed in Somali and was translated to English. We back translated it to Somali and pretested it prior to the interviews with 8 participants. The revised Somali version were finally used for the interviews, as none of the women demanded the English version. Therefore, all interviews were conducted in Somali, which was the native language for both researchers and participants

### Recruitment

The sample frame for ethnic immigrants are available in Norway, but data for a whole ethnic group cannot be accessed, with the aim being to minimize discrimination against minority ethnic groups. Therefore, conventional methods of selecting a random sample for the cross-sectional survey could not be applied. Instead, a snowball sampling was used to identify respondents for the study. Those who filled in the questionnaire were asked to refer other married women in their network to take part in the study. To maximize the representativeness of the sample, different neighborhoods in Oslo, as well as different schools and social gathering places frequented by Somalis, were identified using the local knowledge of the research team. Interviews were conducted at different community gathering places such as mosques, schools, homes and cafeterias based on participants’ preferences. Interviews were conducted by the second author who is a female with Somali descent.

Within Somalia, the security situation in Mogadishu made randomization impossible for researchers, and therefore we used snowball sampling for the recruitment. In both settings, the researchers made every effort to explain the research question in a culturally sensitive way to respondents. Participants were informed about the anonymity of their information, and they were informed to withdraw the study at any time if they wish so. The information letter and consent statement were submitted to REK prior to the interview and documents were approved. The study was ethically approved by the Somali National University and REK.

### Analysis

Data was analyzed using SPSS version 25. We performed a chi-square test for the analyses of categorical variables and a *t*-test for continuous variables. In order to determine the association between exposure and outcome variable, a multivariate logistic analysis was performed. The association was assessed using a 95% confidence interval (CI) and an odds ratio (OR), with the level of significance being determined at a *P* value < 0.05.

## Result

The women in Mogadishu were much younger than the women in Oslo (26.5 vs 34.5) (Table 1). Majority of women in Oslo have lived in Norway over 14 years (39%), while only 8% have lived in Norway 4 years or less. The mean number of pregnancies were not significantly different between the two groups. However, the mean intended fertility was 4 among the Somali immigrant women in Oslo and 10.8 among Somali women in Mogadishu. This difference was significant (p = 0.000). Similarly, significant difference was found in the number of children alive among women in Oslo (3.5 children per women) and women in Mogadishu (4.1 children per women) (p = 0.013).

**Table 1:** Fertility preferences of study participants.

Indicators	Norway	Somalia	P value
	Mean		
Intended fertility	4.3	10.8	0.000
Current fertility (Number of pregnancies)	4.3	5.1	0.132
Children alive	3.5	4.1	0.013
Age	34.5	26.5	0.000

About 17% of study participants in Oslo and 83% of women in Mogadishu had no formal education. Similarly, there was significant difference in attitudes toward contraception between the two groups (P = 0.000). While 86% of participants in Mogadishu and 13.4% of Somali women in Oslo had the belief that contraception is irrelevant for women’s health. The main reason of not using contraception among women in Mogadishu was religious reasons, while side-effects, access and cost of the contraception were the main reasons presented by Somali women in Oslo. Nearly 50% of women in Oslo and those in Mogadishu had unintended childbirth on one occasion or more (Table 2).

**Table 2:** Group differences by country of residence.

Indicators	Norway N (%)	Somalia N (%)	P value
**Education**			
Secondary and higher	83 (84.7)	15 (15.3)	P = 0.000
Primary	106 (76.8)	32 (23.2)	
No formal Education	37 (17.1)	180 (82.9)	
**Occupation**			
Permanent job	45 (78.9)	12(21.1)	P = 0.000
Temporary job or job training	136 (87.7)	19(12.3)	
Unemployed	33 (14.4)	196(85.6)	
**Unintended child birth**			
No	127(49.8)	128(50.2)	P = 0.509
yes	88(49.4)	90(50.6)	
**How do you see contraception**			
Important for women’s health	197(73.8)	70(26.2)	P = 0.000
Not important for women’s health	23(13.4)	149(86.6)	
**If know modern contraception**			
yes	186 (70.7)	77(29.3)	P = 0.000
No	38 (20.8)	145(79.2)	
**Reason for not using contraception**			
Religion	21(23.9)	67(76.1)	P = 0.132
Husband not like it	11(42.3)	15(57.7)	
Want to have a child	26(30.6)	59(69.4)	
Side effect, I cannot find it, expensive	47(37.3)	79(62.7)	

Chi-square test

As shown in Table 3, the unmet-need for contraception was twice as high for Somali women in Mogadishu (48.5) than in Somali women in Oslo (20.2%), as 111/229 and 46/228 women reported unmet needs in Mogadishu and Oslo respectively.

**Table 3:** Unmet needs for contraception.

Indicator	Somalia N (%)	Norway N(%)	Total*
**Are you using contraception now**			
Yes	11 (9)	111 (91)	122
No	202 (65)	108 (35)	310
**Are you pregnant now**			
Yes not sure	17(50)	17 (50)	34
No	210 (50)	208 (50)	418
**Time to wait next pregnancy**			
≤1	75(67)	37(33)	112
2+	143(54)	124(46)	267
**Unmet needs**			
No	103 (38)	169 (62)	272
Yes	111 (71)	46 (29)	157
unmet needs	111/229 = **48.5%**)	46/228 = **20.2%)**	157/457

* Total may not add up due to missing data

Women with no formal education had significantly higher prevalence of unmet needs compared to those with secondary or higher education levels (P = 0.001). Further, participants who had no knowledge about modern contraception were more likely to have unmet needs compared to those who had knowledge about modern contraception (Table 4). Moreover, those who consider modern contraception irrelevant for women’s health had higher prevalence of unmet needs compared to those who see it necessary for women’s health. This difference was statistically significant (P = 0.001).

**Table 4:** Correlates of unmet needs.

Indicators	No	Yes	P value
**Education**			
Secondary and above	69 (73)	25 (26.6)	
Primary	93 (70.5)	39 (29.5)	0.001
No formal education	108 (54.3)	91 (45.7)	
**Occupation**			
Permanent job	38 (70.4)	16 (29.6)	
Temporary job	114 (77.6)	33 (22.4)	P = 0.000
Unemployed	109 (51.2)	104 (48.8)	
**Age**			
≤23	43 (52.4)	39 (47.6)	P = 0.06
24–28	58 (62.4)	35 (37.6)	
≥29	168 (66.9)	83 (33.1)	
**Knowledge of contraception**			
Yes	182 (73.1)	67 (26.9)	P = 0.000
No	85 (49.7)	86 (50.3)	
**Attitudes: How do you see modern contraception**			
important for women’s health	181 (70.4)	76 (29.6)	P = 0.001
Not important at all or not sure	86 (54.5)	72 (45.6)	
**Communication with husband about contraception**			
Yes	130 (74.7)	44 (25.3)	P = 0.001
No	128 (54.2)	108 (45.8)	
**Intended fertility**			
≤3 children	47 (75.8)	15 (24.2)	
4–6 children	91 (74)	32 (26)	P = 0.000
≥7 children	109 (51.9)	101 (48.1)	

Chi-square test

The education-adjusted logistic regression model (Table 5) showed that the odds of having unmet needs for contraception was, nearly, three times higher among Somali women in Mogadishu compared to Somali women in Oslo (OR: 2.6, CI: 2.56–7.68). Women who consider modern contraception irrelevant for women’s health were 1.6 times higher odds of unmet needs for contraception than their counterparts (OR: 1.6, CI: 1.03–3.57). Further, women who do not engage in communication about the contraception with their husbands had two times higher odds of unmet needs for contraception compared to those who do otherwise (OR:2.1 CI:1.31–3.41).

**Table 5:** Factors associated with unmet needs for contraception.

Indicators	Model 1 (crude)	Model 2 (education adjusted)
**Country**		
Norway	1.00	1.00
Somalia	OR: 4.0, CI:2.59–6.03	**OR 2.6, CI:2.56–7.68**
**Knowledge on contraception**		
Yes	1.00	1.00
No	OR: 2.75, CI: 1.82–4.14	OR:2.34, CI:1.50–3.62
**Attitude toward contraception**		
Important for women	1.00	1.00
Not important	OR: 2.0. CI: 1.32-3-01	OR:1.6, CI:1.03–3.57
**Intended fertility**		
≤3	1.00	1.00
4–6	OR: 1.10, CI: 0.54-2-23	OR: 1.0, CI: 0.51-2-15
≥7	OR: 2.9, CI: 1.52–5.51	OR: 2.4, CI: 1.18-4-84
**Communication with the husband**		
Yes	1.00	1.00
No	OR: 2.5, CI: 1.62–3.82	OR:2.1 CI:1.31–3.41

## Discussion

This study is first of its kind that estimated the prevalence of unmet need for contraception and associated indicators among Somali immigrant women in Norway compared with their original population in Somalia. The prevalence of unmet need for contraception among Somali immigrant women in Oslo was 20.2%, which is over two times higher than the unmet needs in Norway and other Scandinavian countries [23]. Unmet need is an independent predictor of unintended pregnancies [24]. Tremendous effort has been made by the Norwegian Government to increase immigrant women’s access, acceptability and utilization of contraceptive methods. But, to counter act traditions, religious beliefs and misperceptions toward modern contraception require tailored intervention, and this poses a formidable challenges to service providers. A growing body of research indicates that the beliefs, attitudes and behaviors regarding contraceptives, and reproductive behaviors are sanctioned by their country of origin [25]. This highlights the value of cross-country research which includes the country of origin. Given the fact that Somalia has one of the lowest contraception prevalence in the world (<10%), it is not unexpected that Somali immigrant women in Norway have higher unmet needs for contraception compared to general Norwegian population. Similar situation has also been found in many other countries with substantial number of Somali immigrants. For example, a prior study in Finland reported that 73% of Somali women do not use contraception for religious or gender related reasons [12]. Another study in the USA identified religious teaching, status of women and an oral tradition as barriers to contraceptive services among Somalis [26]. Majority of Somali refugee women in Kenya were also found not to use contraceptives, due to the numerous barriers they face including religious reasons and partner related issues [27]. However, the fact that nearly 90% of Somali immigrant women consider modern contraception as necessary for women’s health, and that unmet need for contraception amongst Somali women in Oslo is half that of women in Mogadishu, are indications of the substantial success in changing negative attitudes toward contraception among Somali women. Somali immigrants come from a context where contraception is openly rejected by a diverse range of gate keepers, including medical doctors, prominent religious leaders and community elders. The current study and others suggest the need to provide not only family planning, but reproductive health services as a whole within a broader social and cultural dimension that meets the need of Somali women, which entails viewing the contraceptive services within their social and cultural context [26–28].

The study shows a mean number of nearly four children per woman among Somali immigrant women which agrees with the rate reported by the Norwegian statistics central bureau [22]. Similarly, fertility intention of four children per women were found among Somali women in Oslo. The lower fertility intention of Somali women in Oslo compared to their counterparts in Mogadishu could be influenced by the prevalent fertility norms in Norway. A recent study among Somalis in Minnesota reported that host country factors had influenced the fertility desire of Somali women [29]. Somali women who participated in our recent qualitative study admitted the social and economic difficulties associated with raising many children in Norway, which motivated them to use contraceptive methods. Unlike immigrant women in Oslo, fertility preferences of 10.8 children per women was found among Somali women in Mogadishu. Evidences show, in Somalia, a woman’s status is enhanced by her ability to produce numerous children [17]. A qualitative study we conducted both in Oslo and in Mogadishu showed that if a Somali woman does not reproduce children, the husband is likely to abandon her for another, which means reproduction of children is not only family treasure but also a means for women to secure their marriage [28]. In the polygamous life, there is a perception that the woman with the largest number of children can take over the love of the husband. Thus the more the children, the stronger and safer the marriage.

The result show that about 50% of Somali immigrant women in Oslo had experienced unplanned child birth, which questions whether the fertility rate of four, which was observed among Somali women in Norway, are all planned or there are number of unplanned or unwanted childbirths that women had no means to prevent. While all options to prevent unwanted childbirth are available in Norway, including induced abortion, for Somali women, abortion may not be a desired option for religious reasons. Therefore, if there is a limited access to contraceptive methods, the result may be a high prevalence of unintended childbirth, which may contribute to a high fertility rate among Somali immigrant women. This may also impact on the couple’s potential for education and employment, and ultimately the whole integration process. Therefore, we recommend an interventions to increase contraception use of Somali immigrant couples in Norway, and such intervention should target both men and women and to address gender related issues surrounding decision making.

Poor knowledge and negative attitudes toward contraception are associated with unmet needs for contraception. This result is concordant with prior studies, which have found that contraceptive practices and attitudes of immigrant women differed from the perceptions of the mainstream community, for example deep suspicion of hormonal contraception [30]. Similarly, a study in Canada reported that immigrant women expressed a deep suspicion towards hormonal methods of contraception, and were reluctant to use them [31, 32]. Individuals’ knowledge of modern contraceptive methods and positive attitudes towards these methods may increase the acceptance and the uptake of contraceptives while decreasing the related risks such as unplanned childbirth and abortion. A study in Denmark found that immigrant women who lack knowledge on contraception had a 6-fold increased odds for unintended pregnancies and abortion [8], which is ‘more or less’ in line with our finding.

Our study found that women who do not communicate with their partners about the contraception have higher odds of unmet need for contraception. This is in line with prior research that found a strong positive impact of spousal communication on contraceptive use, even when controlling for confounding variables [33]. Our findings show that 20% of women in Oslo have never discussed contraception with their husbands. A prior study in Ghana reported that women who take health decisions with their partners are more likely to use modern contraception as compared to women who take health decisions alone [34]. In several sub-Saharan African countries, partners’ discussion reported to have strong and positive association with women’s contraceptive use [34–36]. Reproductive rights include the right of women to decide how many children they want to have, full access to family planning information and services and modern forms of contraception [37]. Information education and communication (IEC) campaigns geared to partner communication regarding contraception and fertility, and promoting male involvement in family planning could increase contraceptive prevalence and reduce unmet needs for contraception among Somali partners in Oslo and in Mogadishu.

Not having formal education was associated with unmet needs for contraception. This finding is in agreement with prior finding in Kenya where women with lower educational level were unlikely to use modern contraceptives [38]. Level of education of women were also associated with unmet needs and unintended pregnancies [24]. Fertility and contraceptive use in communities in Sub-Saharan Africa are associated with various markers of socioeconomic status, most prominent of which is women's education [38]. In Somalia, like many other developing countries women have a considerably lower social status and autonomy than men and improving women’s education could be one way of improving social status, and ultimately women’s autonomy to their decisions regarding contraception. A research suggests that without increasing women’s education level, the universal access to contraceptive services may not be achieved [39].

The study has potential limitations. One important limitation of this study is its cross-sectional design, hence making it difficult to establish the causes. Moreover, most of the variables were self-reported, with a distinct possibility of both under- and/or over reporting. Further, despite stringent efforts to make the sample as representative as possible, our sampling strategy was likely to result in a sample with some biases. In the process of data collection, there were large number of women who refused to answer the questions in both settings, but majority in Mogadishu. Our sample might therefore over-represent those willing to discuss the contraceptives. Possible biases in the research results arising from this are difficult to assess but we think the most likely bias would be an underestimation of the prevalence of unmet needs for contraception.

## Conclusion

Unmet needs for contraception reported from Somali immigrant women in Oslo is similar to unmet needs in many sub-Saharan African countries. This indicates that both the culture of immigrants’ country of origin and their immigration experience may shape attitudes and practices relevant to reproductive health and it may mediate immigrants’ response to knowledge creation campaigns. Understanding of immigrants’ cultural background, and provision of tailored information is essential for immigrants’ uptake of contraception methods. Therefore, training primary health providers for provision of tailored information about the modern contraception to immigrant women, which includes an individualized counselling may improve partners’ knowledge, demand and uptake of modern contraception. Further, in order to meet the need of immigrant women for modern contraception and to ensure the rights to Planned Parenthood, tailored interventions in the form of Information Education Communication to both men and women, may reduce the high unmet need for contraception and also improve partner communication on family planning among Somali immigrants in Oslo. A future research is required to assess reproductive health access and utilization of diverse immigrant communities in Norway.
